# Estimated Radon Exposure in Eastern Pennsylvania Schools

**DOI:** 10.1001/jamanetworkopen.2024.48676

**Published:** 2024-12-03

**Authors:** Brian Yang, Chrysan Cronin, Beth A. Tarini

**Affiliations:** 1 Moravian Academy, Bethlehem, Pennsylvania; 2 Department of Public Health, Muhlenberg College, Allentown, Pennsylvania; 3 Children’s National Research Institute, Children’s National Hospital, Washington, DC; 4 School of Medicine and Health Sciences, George Washington University, Washington, DC

## Abstract

This cross-sectional study examines publicly available data to estimate school-based radon and radiation dose exposure to children attending school in 5 eastern Pennsylvania districts to estimate children’s annual additional risk as a result of those locations.

## Introduction

Radon 222 is the leading cause of lung cancer in nonsmokers.^1^ Children’s lung cancer risk from radon exposure may be up to 3-fold higher than adults exposed to the same radon amount.^1^ Indoor spaces can have elevated radon levels due to lower particle dispersion.^2^ Pennsylvania has the third-highest US average indoor radon levels.^3^ In Pennsylvania, schools are not required to test for radon, despite Environmental Protection Agency (EPA) recommendations. Radon mitigation reduces indoor radon exposure after radon testing detects elevated radon. The EPA recommends radon mitigation at 4 pCi/L, whereas the World Health Organization recommends mitigation at or above 2.7 pCi/L. We estimated children’s annual additional radon and radiation exposure risk from public schools in 5 eastern Pennsylvania school districts.

## Methods

This cross-sectional study followed the STROBE reporting guideline. Because we used publicly available deidentified data, no IRB review or patient consent was required. We used average indoor residential basement radon levels (AIRRL) data with spatial resolution based on zip codes to estimate school-based radon and radiation dose exposure to children attending five eastern Pennsylvania school districts. Pennsylvania Department of Environmental Protection (PADEP) collected these data from 1990 to 2022.^4^ Using National Center for Educational Statistics district data,^5^ we matched the school’s zip code to PADEP average basement radon data for that zip code.

To provide clinical context of annual school-based radiation dose, we converted the PADEP average indoor radon level for each zip code from picocurie per liter (pCi/L) to millisieverts (mSv) and millirem (mrem) (eAppendix in Supplement 1). We calculated the projected radiation exposure in mSv and mrem using the 2018 Protection Summary of International Commission on Radiological Protection (ICRP) Recommendations on Radon. We conservatively estimated annual school-based radon radiation exposure based on 1200 hours/school year.^2^ The statistical analysis was performed from April 21 to June 4, 2024.

## Results

Based on our analyses, all children attending public schools in these 5 eastern Pennsylvania school districts (n = 39 195) may have radon exposure that exceeds the EPA recommended action level of 4.0 pCi/L (Table, Figure). Notably, 7651 children (19.5%) were found to attend public schools in zip codes where the AIRRL exceeded 11.0 pCi/L; within this highly exposed group, 3373 (44.1%) were elementary school students. Because of elevated school radon levels, these students may be exposed to additional potential annual radiation dose ranging from 1.37 mSv (136.84 mrem) to 5.98 mSv (598.93 mrem).

**Table.** Estimated Radon Exposure in 5 Northeastern Pennsylvania School Districts

Zip code	No. of students^a^	School type and No. (No. of students)			Mean radon level, pCi/L^b^	Estimated annual radiation exposure, mSv (mrem)^c^
		Elementary	Middle	High school		
**Allentown School District (n = 15 938)**						
18013	2977	1 (636) 2 (524) 3 (278) 4 (532)	1 (1007)	NA	7.1	2.11 (211.21)
18104	4907	1 (488) 2 (658)	1 (909)	1 (2852)	6.9	2.05 (205.26)
18102	4515	1 (813) 2 (662) 3 (240) 4 (625) 5 (560) 6 (356)	1 (820)	1 (439)	5.3	1.58 (157.66)
18109	2806	1 (482) 2 (409)	NA	1 (1915)	5.2	1.55 (154.69)
18101	733	NA	1 (733)	NA	4.6	1.37 (136.84)
**Bethlehem Area School District (n = 12 960)**						
18015	1462	1 (513) 2 (449)	1 (500)	NA	7.0	2.08 (208.24)
18017	3808	1 (339) 2 (347) 3 (459) 4 (226) 5 (263) 6 (319) 7 (300) 8 (508)	1 (1047)	NA	6.7	1.99 (199.31)
18020	2114	1 (361)	NA	1 (1753)	6.6	1.96 (196.34)
18045	361	Elementary School 1 (361)	NA	NA	6.5	1.93 (193.36)
18018	5215	1 (224) 2 (382) 3 (224) 4 (239)	1 (781) 2 (725)	1 (2640)	5.8	1.73 (172.54)
**Northampton School District (n = 5249)**						
18088	492	1 (492)	NA	NA	19.3	5.74 (574.14)
18014	831	1 (384) 2 (447)	NA	NA	17.1	5.09 (508.69)
18067	3926	1 (808)	1 (1256)	1 (1862)	11.2	3.33 (333.18)
**Northwestern Lehigh School District (n = 1971)**						
18066	1583	1 (423)	1 (465)	1 (695)	20.1	5.98 (597.93)
19530	388	1 (388)	NA	NA	18.7	5.56 (556.29)
**Southern Lehigh School District (n = 3077)**						
18036	431	1 (431)	NA	NA	20.1	5.98 (597.93)
18034	1954	1 (432)	1 (466)	1 (1056)	7.1	2.11 (211.21)
18015	692	1 (692)	NA	NA	7.0	2.08 (208.24)

Abbreviation: NA, not applicable.

^a^ Data adapted from the Institute of Education Sciences Search for Public School Districts tool.^5^

^b^ Data adapted from the Pennsylvania Department of Environmental Protection RadonZip report viewer.^4^

^c^ Data were derived from the International Commission on Radiological Protection summary of recommendations on radon.^2^

**Figure.**
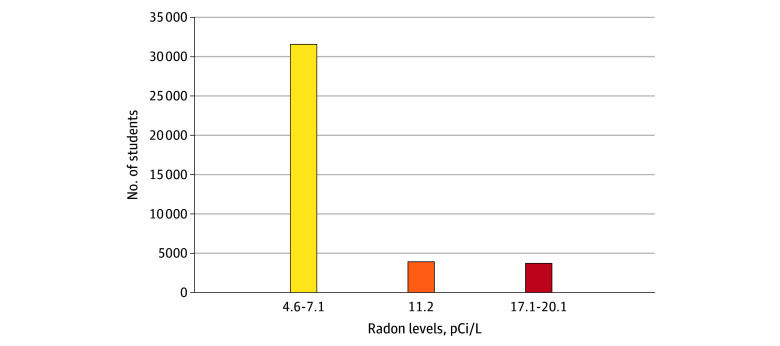
Estimated Radiation Exposure The Environmental Protection Agency–recommended level at which action is undertaken to reduce radon levels is 4.0 pCi/L.

## Discussion

We found that all children in the 37 public schools in 5 eastern Pennsylvania school districts attend a school where average residential zip code radon levels exceed the EPA recommended action level of 4.0 pCi/L. As a result, some of these students may be exposed to approximately twice the total annual dose from natural background radiation, which the US Nuclear Regulatory Commission estimates is approximately 3.1 mSv (310 mrem).^6^

Although this study’s limitations include using radon levels collected from residential homes between 1990 and 2022, meaningful radon exposure differences between households and schools based on level heights, time of exposure, and building structures (number of floors) are minimal. Other parameters, such as breathing rate or the number of hourly air exchanges in the structure, may increase or decrease individual radon exposure. This analysis does not include potential for tobacco coexposure and radon exposure at home, both of which increase health effects of radon. Despite its limitations, this study underscores the urgent need to conduct radon testing in schools and, if necessary, mitigate children’s exposure to school-based radon. Cumulative radiation exposure from radon in unmitigated school settings may have significant short- and long-term health effects on children because of the radiosensitivity of their developing organs and tissues. Moreover, children remaining in these school districts for their entire education could be exposed to up to 12 years of elevated radon.

## Notes

Supplement 1.
eAppendix.

eReference.

Supplement 2.
Data Sharing Statement
