# Supplementary material for: Estimated Radon Exposure in Eastern Pennsylvania Schools
Source: JAMA Netw Open. 2024 Dec 3;7(12):e2448676. doi: 10.1001/jamanetworkopen.2024.48676 (PMC11615708; doi:10.1001/jamanetworkopen.2024.48676)
Supplement: Supplement 1. — eAppendix. eReference. [file jamanetwopen-e2448676-s001.pdf]

Supplemental Online Content

Yang B, Cronin C, Tarini BA. Estimated radon exposure in eastern Pennsylvania schools. *JAMA Netw Open*. 2024;7(12):e2448676.
doi:10.1001/jamanetworkopen.2024.48676

eAppendix.

eReference.

This supplemental material has been provided by the authors to give readers additional information about their work.

© 2024 Yang B, et al. *JAMA Network Open*.

eAppendix.

To calculate effective radiation dose, multiply the appropriate coefficient by the radon (or radon progeny) concentration and the time exposed.[1]

For example: Dose Coefficient ($mSv/Bq\ h\ m^{-3}$) × Radiation (Bqm^{-3}) × Time Exposed (hr) = *x* mSv

eReference.

1. International Commission on Radiological Protection. Summary of ICRP recommendations on radon. ICRP ref 4836–9756–8598. January 26, 2018. Accessed May 31, 2024. http://www.icrpaedia.org/images/f/fd/ICRPRadonSummary.pdf

© 2024 Yang B, et al. *JAMA Network Open*.
