# Supplementary material for: Estimated Radon Exposure in Eastern Pennsylvania Schools
Source: JAMA Netw Open. 2024 Dec 3;7(12):e2448676. doi: 10.1001/jamanetworkopen.2024.48676 (PMC11615708; doi:10.1001/jamanetworkopen.2024.48676)
Supplement: Supplement 2. — Data Sharing Statement [file jamanetwopen-e2448676-s002.pdf]

Data Sharing Statement

Yang. Estimated Radon Exposure in Eastern Pennsylvania Schools. *JAMA Netw Open*. Published December 03, 2024. doi:10.1001/jamanetworkopen.2024.48676

Data
Data available: Yes
Data types: Data (not involving human participants)
How to access data: http://cedatareporting.pa.gov/Reportserver/Pages/ReportViewer.aspx?/Public/DEP/RP/SSRS/RadonZip https://nces.ed.gov/
When available: With publication

Supporting Documents
Document types: None

Additional Information
Who can access the data: Anyone requesting the data
Types of analyses: Calculation of Effective Dose from ICRP Summary
Mechanisms of data availability: With Investigator Support
